# Endemic and cosmopolitan fungal taxa exhibit differential abundances in total and active communities of Antarctic soils

**DOI:** 10.1111/1462-2920.14533

**Published:** 2019-02-22

**Authors:** Filipa Cox, Kevin K. Newsham, Clare H. Robinson

**Affiliations:** ^1^ School of Earth & Environmental Sciences The University of Manchester Manchester M13 9PL UK; ^2^ NERC British Antarctic Survey, High Cross, Madingley Road Cambridge CB3 0ET UK

## Abstract

Our understanding of the diversity and community dynamics of soil fungi has increased greatly through the use of DNA‐based identification. Community characterization of metabolically active communities via RNA sequencing has previously revealed differences between ‘active’ and ‘total’ fungal communities, which may be influenced by the persistence of DNA from nonactive components. However, it is not known how fungal traits influence their prevalence in these contrasting community profiles. In this study, we coextracted DNA and RNA from soil collected from three Antarctic islands to test for differences between total and active soil fungal communities. By matching these geographically isolated fungi against a global dataset of soil fungi, we show that widely dispersed taxa are often more abundant in the total community, whilst taxa restricted to Antarctica are more likely to have higher abundance in the active community. In addition, we find that active communities have lower richness, and show a reduction in the abundance of the most dominant fungi, whilst there are consistent differences in the abundances of certain taxonomic groups between the total and active communities. These results suggest that the views of soil fungal communities offered by DNA‐ and RNA‐based characterization differ in predictable ways.

## Introduction

Soil fungi have pivotal roles in terrestrial ecosystems as decomposers, pathogens and partners in mycorrhizal and lichen symbioses. Molecular techniques have substantially increased our knowledge of fungal communities in soils across the globe (Tedersoo *et al*., 2014), particularly with the onset of next‐generation techniques, which preclude the use of a cloning step and have allowed the direct sequencing of soil fungal nucleic acids. Almost all studies carried out to date have relied on the sequencing of soil fungal DNA, which is recalcitrant and relatively long‐lived in the environment (Willerslev *et al*., 2007). As a result, these studies are likely to have sequenced DNA extracted from inactive sources, such as spores, dead hyphae and extracellular DNA (van der Linde and Haller, 2013), with a recent study estimating that the latter can account for up to 40% of total DNA recovered from soils (Carini *et al*., 2016). By measuring a soil's ‘total’ fungal community (i.e., that derived from its DNA profile), it is not possible to distinguish fungi that are active in processes such as decomposition from those that may represent relic or dormant fungal communities, and it is possible that descriptions of richness and relative abundances of taxa are consequently skewed (Carini *et al*., 2016).

Soil fungal community composition can also be determined through sequencing of the precursor rRNA internal transcribed spacer (ITS) region, a much shorter‐lived molecule than DNA, which is present predominantly in metabolically active tissue (Anderson and Parkin, 2007). Whilst this technique has been used in relatively few studies focused on fungi, it has been shown that a soil's ‘active’ fungal community (i.e., that derived from its RNA profile) may have lower richness than the community measured through DNA‐based analyses, and certain taxa may show markedly different abundances (Taylor *et al*., 2010; Rajala *et al*., 2011; Baldrian *et al*., 2012; van der Linde *et al*., 2012; Romanowicz *et al*., 2016). Despite this disparity, studies have not yet attempted to determine how fungi possessing different life history traits might be represented in total and active soil communities. If community descriptions based on analyses of DNA are biased towards specific taxa displaying similar traits, our understanding of fungal communities across the globe may be skewed, in a manner analogous to the biases inherent in culture‐based community descriptions, which select for generalist species capable of growing on artificial media under laboratory conditions (Bridge and Spooner, 2001).

We have previously demonstrated a striking bimodal distribution of range sizes among Antarctic soil fungi (Cox *et al*., 2016), showing the presence of endemic taxa – which is to be expected, owing to the region's geographical isolation – and a large number of cosmopolitan fungi with global range sizes. Fungi with such wide distributions may be habitat generalists, although the presence of bipolar fungi (occurring at high latitudes in both hemispheres, but not in between) suggests that at least some are cold‐adapted taxa. This disparity in range sizes may be owing to different dispersal strategies, and represents an opportunity to test for the contributions of endemic and cosmopolitan fungi to the total and active fungal communities of geographically isolated maritime Antarctic soils. Gaining a better understanding of the active fungal community of these soils is also imperative, since they are likely to contain significant stocks of old, recalcitrant carbon fractions (Newsham *et al*., 2018), and decomposition processes in them are thought to be dominated by fungi (Yergeau *et al*., 2007).

**Figure 1:**
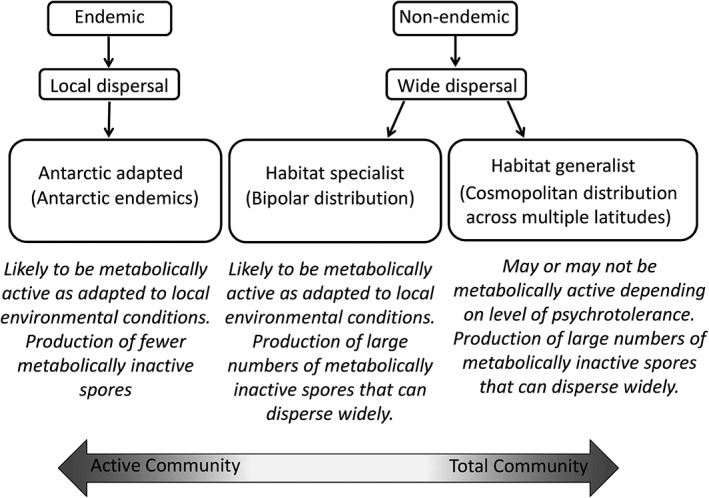
Conceptual framework in which habitat specialization and dispersal of fungal taxa might predict their abundance in either the active or total communities of Antarctic soils. See main text for further explanation.

Here, we develop a conceptual framework (Fig. 1), in which the relative abundances of fungal taxa in total and active Antarctic soil communities is based on their presence across global sites and in Antarctic soils. We predict that endemic fungi found exclusively in Antarctica will be more abundant in the active community, because of the adaptations of these species to local environmental conditions (Imbert *et al*., 2011; Berdahl *et al*., 2015), such as the aridity, low temperatures (< 0 °C for c. 8 months per annum and annual minima to −40°C) and wide temperature fluctuations (annual ranges 35–65°C) encountered in maritime Antarctic soils (Convey *et al*., 2018). At the other extreme, we anticipate that cosmopolitan fungi identified as occurring in soils across the globe will be relatively less abundant in active Antarctic soil communities, because of their poorer adaptations to local conditions (Büchi and Vuilleumier, 2014). In addition, compared with endemic fungi, cosmopolitan fungi with large range sizes may produce greater numbers of dormant spores containing little RNA relative to DNA (van der Linde and Haller, 2013). Between these two extremes, we hypothesize that bipolar fungi are likely to produce copious spores capable of dispersal over large distances, replicating the dispersal strategy of generalist species and mirroring the long‐distance dispersal of some specialist plant and animal species between suitable habitat patches (Spiegel and Nathan, 2007; Centeno‐Cuadros *et al*., 2011). However, as habitat specialists, these fungi should be capable of active growth and metabolism in Antarctic soils, so on balance may show a more even abundance across the DNA and RNA pools, depending on the relative strength of these two drivers.

In the present study, we characterize the active and total fungal communities present in soils from three geographically isolated maritime and sub‐Antarctic islands. Fungal community profiles derived from analyses of soil RNA and DNA are compared to explore how they differ in terms of species richness, community evenness, community and taxonomic composition, as well as how the magnitude of these differences changes with soil depth, which has been found to influence the abundance of fungi in numerous habitats (Dickie *et al*., 2002; Lindahl *et al*., 2007), and of endemic Archaea in fumarolic soils of Antarctica (Herbold *et al*., 2014). By comparison with a global dataset of fungi (Cox *et al*., 2016), we assign the fungi identified in Antarctic soils as putatively endemic, cosmopolitan or bipolar, to test whether patterns of abundance match those predicted by traits in the conceptual framework shown in Fig. 1.

## Results

### *Sequence data and taxonomic assignments*

**Figure 2:**
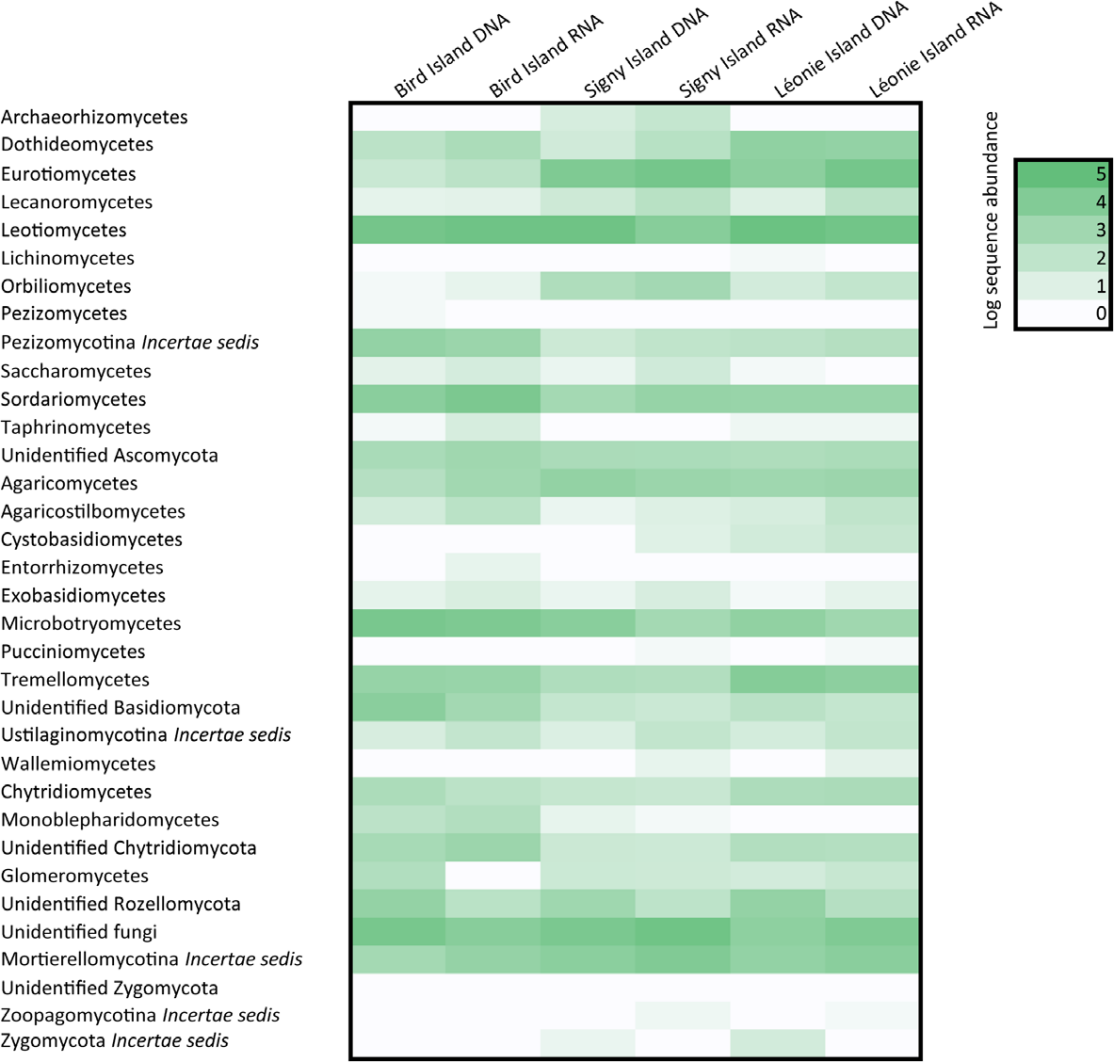
Heatmap showing the relative proportion of sequences belonging to different taxonomic classes, sub‐phyla or phyla in the total and active soil fungal communities at the three Antarctic islands. Darker shades indicate higher relative proportions of taxonomic groups. [Color figure can be viewed at http://wileyonlinelibrary.com]

Across the three Antarctic islands, we obtained a total of 1 181 155 raw sequences from RNA, to combine with the 932 024 sequences previously described from DNA samples. After quality filtering, ITS extraction and removal of chimeric, non‐fungal or singleton sequences, 1 114 807 and 853 646 sequences remained in the RNA and DNA datasets respectively. There was variation in the number of sequences remaining per island, with the greatest number of sequences being obtained for RNA in Bird Island soil (total of 386 029), and the lowest for DNA in Signy Island soil (total of 226 472). The abundances of all taxa varied across islands and between total and active communities (Fig. 2). Overall, the Leotiomycetes were the most frequently sequenced class, with members of the Helotiales being the most commonly sequenced order. Members of the Eurotiomycetes, Sordariomycetes and Microbotryomycetes were also frequent in soil (Fig. 2).

### *Abundances of endemic, cosmopolitan and bipolar fungi in active and total communities*

**Figure 3:**
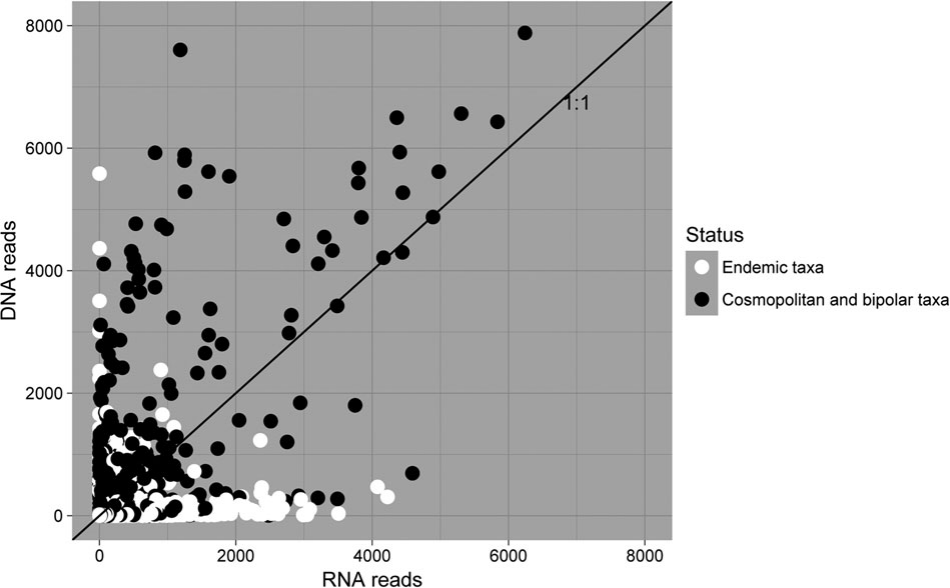
The numbers of RNA and DNA reads of each OTU, in each sample in which it was recorded, as a function of each other. Note that OTUs endemic to Antarctica are more abundant in the active fungal community (i.e., occur below the 1:1 line) and that OTUs which are cosmopolitan are more abundant in the total fungal community (i.e., occur above the 1:1 line). To allow valid comparisons of species abundance whilst controlling for the total number of reads, the total number of reads per sample was rarefied to the lowest common sequencing depth.

**Figure 4:**
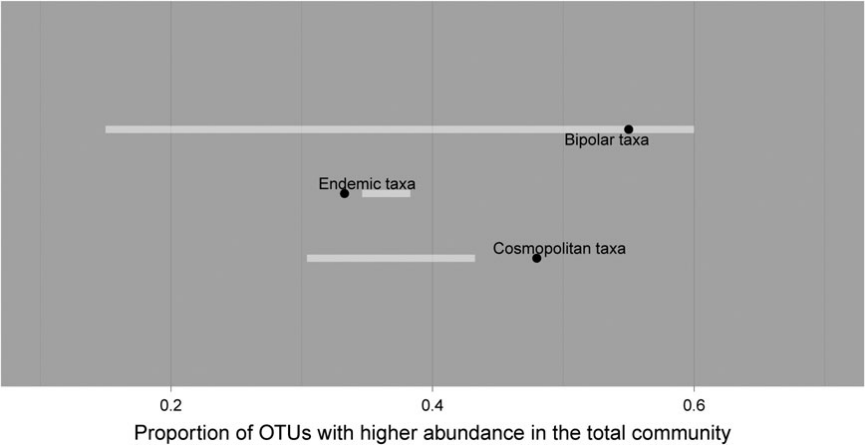
Results of the permutation paired sign test. Filled circles and white bars show observed values and 95% expected ranges respectively. There was a lower than expected proportion of endemic taxa with higher abundance in the total community, whilst the proportion of cosmopolitan taxa with higher abundance in the total community was greater than expected.

Endemic and cosmopolitan fungi showed differential patterns of abundance between the active and total communities (Fig. 3). Fungal OTUs identified as being endemic to Antarctica were significantly more likely to have higher abundance in the active fungal communities (Fig. 4, *P* < 0.001, observed proportion = 0.667, expected 95% confidence set: 0.617–0.654). In contrast, OTUs of cosmopolitan fungi were significantly more likely to have higher abundance in the total community than would be expected by chance (Fig. 4, *P* < 0.001, observed proportion = 0.480, expected 95% confidence set: 0.304–0.432). Bipolar fungi tended towards having higher abundances in the total community, but this difference was not significant (Fig. 4, *P* = 0.077, observed proportion = 0.550, expected 95% confidence set = 0.150–0.600).

### *Richness, evenness and taxonomic distinctness of total and active soil fungal communities*

**Figure 5:**
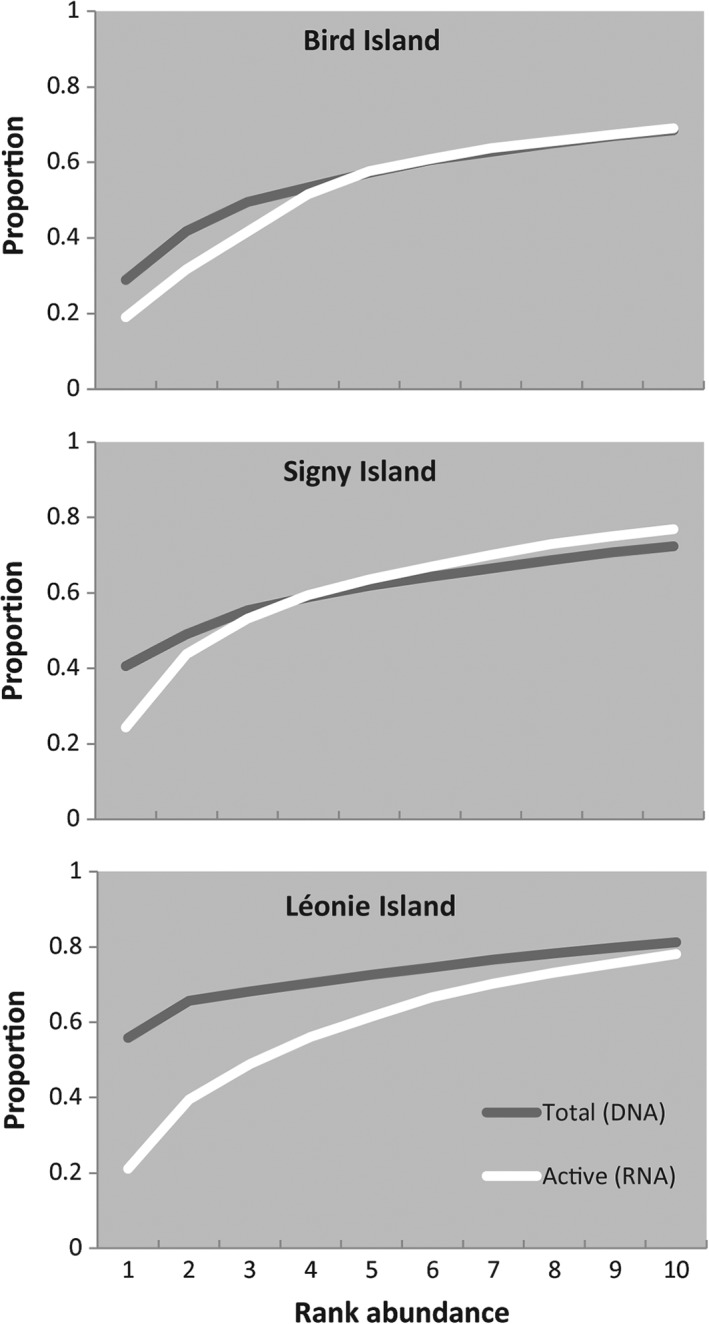
K‐dominance plots of total and active soil fungi communities at the three Antarctic islands. The cumulative proportion of the overall community made up by the 10 most abundant OTUs on each island are shown, to illustrate the lower abundance of the dominant fungi in the active communities.

There was a positive correlation between the richness of fungal OTUs found within the DNA and RNA pools in each soil sample (for 1000 iterations of the rarefied datasets, 95% confidence set of *r* = 0.629–0.669; a plot of one random iteration is presented in Supporting Information Fig. S1). Across the three islands, significantly fewer fungal OTUs were identified in the RNA pool than in the DNA pool from the same sample (Table 1). Although the difference was marginally nonsignificant (*P* = 0.0501), species evenness of RNA was consistently higher than that of DNA at each of the individual islands (Table 1), and species distribution curves show lower abundances of the most dominant fungi in the RNA libraries (Fig. 5). Most of the more abundant OTUs were present in both total and active communities. Overall, around 24% of OTUs in the un‐rarefied dataset were only present in the total community, and 22% were only present in the active community, although these tended to be rarer OTUs. Taxonomic distinctness did not differ between the two libraries on any of the three islands (Table 1).

**Table 1:** Differences in diversity indices between total (DNA‐derived) and active (RNA‐derived) soil fungal communities.

		Bird Island^†^			Signy Island^†^			Léonie Island^†^			All islands^‡^		
		Mean	χ^2^	*P* value	Mean	χ^2^	*P* value	Mean	χ^2^	*P* value	Mean	χ^2^	*P* value
Richness	DNA	191.44–191.67			146.67–148.00			120.78–122.00			153.15–153.74		
			11.43	< 0.001		11.57	< 0.001		0.17	0.677		15.08	< 0.001
	RNA	153.89–156.00			116.11–119.00			116.67–119.22			129.41–130.85		
Evenness	DNA	0.47			0.51			0.41			0.46		
			3.30	0.069		0.90	0.344		2.25	0.133		3.84	0.050
	RNA	0.51			0.53–0.54			0.48			0.51		
Taxonomic distinctness	DNA	76.98			77.09–77.13			70.82–70.88			74.97–74.99		
			2.34	0.126		3.62	0.057		0.26	0.609		0.52	0.470
	RNA	75.42–75.47			75.64–75.72			71.91–71.98			74.34–74.37		

Comparisons were made between 1000 iterations of rarefied community matrices – the 95% confidence range of the mean is shown where variation is greater than 3 dp. Tests were carried out between 9^†^ and 27^‡^ pairs of samples.

Across both active and total communities, fungal richness decreased with soil depth, from an average of 147 OTUs at 2 cm to 133 OTUs at 8 cm, but the relationship was not significant (*χ* ^2^ = 2.278, *P* = 0.131), and there was no effect of soil depth on the magnitude of the difference in fungal richness between total and active communities (*χ* ^2^ = 0.334, *P* = 0.564) – i.e., the difference in fungal richness between total and active communities did not change with depth.

### *Compositional differences between total and active soil fungal communities*

Adonis analyses were used to test for differences in the composition of soil fungal communities characterized using either RNA or DNA. Significant differences were detected between the composition of active and total soil fungal communities across all islands (*r* ^2^ = 0.082, pseudo‐*F* = 23.9, *P* < 0.001) and within each of the three islands (Bird Island: *r* ^2^ = 0.131, pseudo‐*F* = 13.312, *P* < 0.001, Signy Island: *r* ^2^ = 0.400, pseudo‐*F* = 58.631, *P* < 0.001, Léonie Island: *r* ^2^ = 0.149, pseudo‐*F* = 15.419, *P* < 0.001), demonstrating that when communities were sampled from the same location, those characterized using DNA were more similar to other samples characterized using DNA than to those characterized using RNA, and vice versa. A generalized linear mixed effect model also showed that the proportion of fungi shared between active and total communities decreased across all islands with increasing soil depth (*χ* ^2^ = 13.378, *P* < 0.001). Tests within each island showed that this effect was present at Signy Island (*χ* ^2^ = 25.774, *P* < 0.001), but not at the other two islands (Bird Island: *χ* ^2^ = 0.876, *P* = 0.349; Léonie Island: *χ* ^2^ = 0.262, *P* = 0.609).

Two classes of fungi showed significantly different patterns of abundance between the total and active communities across the three islands: OTUs belonging to the Leotiomycetes and the Microbotryomycetes more often showed higher abundance in the total community than in the active community (*P* < 0.001).

Abundances of fungal genera are provided in Supporting Information Table S1. Eight OTUs in the Trichocomaceae (members of *Penicillium*, *Paecilomyces* and *Aspergillus*) were consistently recorded as cosmopolitan taxa (Supporting Information Table S1). By contrast, all but three of the 86 OTUs in the Chytridiomycota were found to be endemic to Antarctica (Supporting Information Table S1). The same pattern was observed for lichen‐forming fungi, with all but one of the 44 OTUs in the Verrucariales and the Lecanoromycetes being recorded as endemic to the Antarctic (Supporting Information Table S1).

## Discussion

The data reported here indicate that endemic Antarctic fungal taxa exhibited higher abundances in the active than in the total community of Antarctic soils, and, *vice versa*, that cosmopolitan soil fungi were more likely to occur in the total than in the active community. These observed patterns, which match those predicted by our conceptual framework (Fig. 1), show for the first time that the distribution patterns of microbes can influence their abundances in active and total communities of geographically isolated soils. It is important to note that we have tested only for patterns of abundance consistent with our conceptual framework, and not for the mechanisms responsible for these observed patterns: for example, no quantification of spores, soredia, necromass or other metabolically less active tissues in soils was carried out in this study. A possible explanation for this observation is that endemic taxa are better adapted to the extreme environmental conditions encountered in the soils of the region, although what these specific adaptations are remains unknown. This view is supported by previous research showing that individual isolates of fungi, bacteria and flagellates from Antarctic soils and freshwaters have lower temperature optima for growth than those from tropical and temperate zones (Franzmann, 1996; Boenigk *et al*., 2006; Tojo and Newsham, 2012). However, studies showing that endemic microbes are more active than cosmopolitan taxa in the natural environment through analyses of the relative abundances of RNA and DNA have not, to our knowledge, previously been published in the literature.

Another possible explanation for the observations reported here is that cosmopolitan fungi have a tendency to produce large numbers of spores (which generally have low metabolic activity) in order to achieve long‐range dispersal (Fig. 1), which is consistent with the extremely large latitudinal ranges observed for non‐endemic Antarctic soil fungal taxa (Cox *et al*., 2016). Information on spore formation by fungi in soil, as well as the geographical limits of spore dispersal by specific taxa, is presently very limited. Some fungi with wind‐dispersed spores have been shown to exist as distinct phylospecies on separate land‐masses, indicating geographic barriers to spore dispersal (Shen *et al*., 2002; Geml *et al*., 2008). However, some fungi are suspected to be globally widespread (Pringle *et al*., 2005; Jacquemyn *et al*., 2017), and studies suggest that fungal spores have the potential to travel huge distances, including between South America and maritime Antarctica (Ingold, 1971; Marshall, 1996a; Brown and Hovmøller, 2002). In agreement with our conceptual framework, the data here show that the eight OTUs in the Trichocomaceae (members of *Penicillium*, *Paecilomyces* and *Aspergillus*), which on artificial media produce copious conidia typically measuring < 6 μm × < 4 μm (Domsch *et al*., 1980), were recorded as cosmopolitan taxa in Antarctic soils. By contrast, in a pattern similar to that demonstrated in plants, in which endemic taxa with limited ranges are often characterized by a lower production of seed than is typical for species with wider distributions (Murray *et al*., 2002; Lavergne *et al*., 2004), all but three of the 86 members of the Chytridiomycota, flagellated fungi that typically disperse via zoospores in water films over distances of a few centimetres (Powell and Letcher, 2014), were found to be endemic to Antarctica. Strikingly, the same pattern was found for lichen‐forming fungi, with all but one of the 44 OTUs in the Verrucariales and the Lecanoromycetes being recorded as endemic Antarctic taxa, suggesting that these fungi are inefficient at long‐range dispersal. This observation is consistent with previous reports of endemism in the Antarctic lichen flora (Lee *et al*., 2008; Jones *et al*., 2015) and the finding that the majority of lichen propagules trapped from Antarctica air are not spores, but soredia, measuring 30–60 μm (Marshall, 1996b).

Across all three islands, we consistently found that the richness of the active fungal community was lower than that of the total community, in contrast with previous studies that have found no differences between these two communities (Baldrian *et al*., 2012; Romanowicz *et al*., 2016; Žifčáková *et al*., 2016). This disparity may be driven by increased preservation of relic DNA in the cold soils of Antarctica (Willerslev *et al*., 2004), leading to inflated species counts (Carini *et al*., 2016), or because the relatively low overall richness of soil fungi in this region (Cox *et al*., 2016) results in fewer fungi being available for detection. Despite lower richness at the sample level, a substantial proportion (> 20%) of the fungal taxa identified in the RNA sequencing library were not present in the DNA sequencing library. This may indicate the presence of fungi which, although rare in the community in terms of biomass, may be metabolically very active and functionally significant contributors to ecosystem processes (Deacon *et al*., 2006; Baldrian *et al*., 2012). The active community also showed higher levels of evenness, primarily caused by a reduction in the dominance of the most abundant fungi. The most extreme example of this occurred on Signy Island, where the most abundant OTU, an unidentified member of the Helotiales, represented 40% of the DNA, but just 2% of the RNA, sequences in soil. This reduction in the abundance of dominant fungal OTUs may also partly explain the large number of fungi only detected in the RNA libraries, with the increased available sequencing depth enabling the detection of additional rare active OTUs in the RNA pool.

The composition of total and active communities sampled from the same location were found to significantly differ across and within each island, although the correlation coefficient value for the across‐island test was low, indicating that total compositional differences may be small. We found little difference in taxonomic distinctness between the total and active fungal communities, suggesting that the same taxonomic groups were detected in both nucleic acid libraries, even if the abundances of some groups were very different. However, as a relatively high proportion of these Antarctic fungi could not be identified to even a low taxonomic resolution, we were limited in our ability to test for taxonomic distinctiveness between active and total communities. Two classes, the Microbotryomycetes and the Leotiomycetes, were found in higher abundances in the total compared with the active community. The higher abundance of the Microbotryomycetes in the DNA‐derived community suggests that the frequent occurrence of Basidiomycetous yeasts such as *Rhodotorula* spp. in Antarctic soils (Adams *et al*., 2006; Newsham *et al*., 2016) may not be reflected in their influence on soil functions such as nutrient cycling in the natural environment. The finding that the Leotiomycetes, the most species rich and abundant fungal class in the soils studied here, are frequent in the total community corroborates previous research showing that members of this taxon are widespread in DNA‐based libraries constructed from Antarctic soils (Newsham *et al*., 2016). Members of the Helotiales, one of the most speciose orders of the class, are also frequent in the roots of *Deschampsia antarctica* and *Colobanthus quitensis* (Upson *et al*., 2009), the two plant species from under which soil was sampled in the present study.

Total and active fungal communities both showed a significant shift in composition across soil depths, in agreement with several other studies (Dickie *et al*., 2002; Lindahl *et al*., 2007; Talbot *et al*., 2014). Whilst some of these studies have highlighted how these differences are often largely driven by shifts between mycorrhizal and decomposer fungi across soil horizons, we found strong changes despite an absence of mycorrhiza‐forming fungi in Antarctic soils (Newsham *et al*., 2009), suggesting shifts in composition relating to changes in abundance of other root‐associated fungal taxa such as the Helotiales (Upson *et al*., 2009), and/or because of changing carbon resources across soil depths. This is in agreement with other studies that have shown depth‐related structuring within fungal guilds (Taylor and Bruns, 1999; Taylor *et al*., 2014). We also anticipated that we might detect consistent differences in the extent to which total and active fungal communities differed across soil sampling depths (c.f. Herbold *et al*., 2014), possibly because of deposition of the spores of cosmopolitan fungi onto soil surfaces (Edman *et al*., 2004). However, whilst there was a significant reduction in the proportion of fungal OTUs shared between the two nucleic acid pools at greater soil sampling depths, this pattern was only evident on one of the three islands. This finding may, therefore, be dependent upon local ecosystem processes such as spore deposition rates, or the extent of mixing between soil horizons.

Although the DNA and RNA used for characterizing total and active soil fungal communities in this study were simultaneously coextracted and sequenced using the same methodology, it is appropriate to issue caveats regarding the results of their comparisons. Using sequence read abundance as a proxy for fungal abundance in the natural environment may be inaccurate owing to variation between different taxa in ITS copy numbers, as well as PCR biases arising from variations in the lengths of ITS regions between species (Lindahl *et al*., 2007). Although care was taken to match the denoising and clustering algorithms used for constructing the DNA, RNA and worldwide reference libraries, sequence processing steps such as denoising and OTU clustering can introduce errors or sources of bias. The 97% cut‐off used to group OTUs, although widely used in many mycological studies (e.g., Baldrian *et al*., 2012; Peay *et al*., 2012; Talbot *et al*., 2014), is arbitrary and may fail to differentiate taxa with more conserved ITS regions. In addition, the RNA and DNA extracted from soil were sequenced here on separate plates, which may introduce stochastic differences between the abundance or presence of particular OTUs. There remain gaps in our knowledge regarding how differential stability of DNA and RNA molecules, and how the reverse transcription required for RNA processing, affect community descriptions. However, although all of these caveats should be considered, they would only affect the central question of this study – i.e., whether or not endemic and cosmopolitan fungal taxa exhibit differential abundances in total and active communities – if biases are concentrated in particular taxonomic groups in a way that correlates with differences in endemism and cosmopolitanism.

## Conclusions

Here, we found evidence for the first time that the traits related to fungal distribution patterns can affect their relative abundances within total and active communities of Antarctic soils. These patterns are consistent with a framework in which endemic and cosmopolitan fungi show differences in dispersal strategy, and degree of adaptation to the extreme environment of Antarctic soils, but further investigations are needed to identify the causes of such differences between total and active community profiles. Our analyses also indicated consistent differences in the community composition of fungi in the active and total communities of Antarctic soils, with the increased sequencing depth provided by RNA sequencing seeming to enable the detection of additional fungi. The findings suggest that studies based purely on the total microbial community, assessed by DNA‐based analyses, may be biased towards specific taxa in a predictable manner.

## Experimental procedures

### *Sample collection*

Between October and November 2011, soil samples were collected from Bird Island (54.0089°S, 38.0662°W), Signy Island (60.7107°S, 45.5849°W) and Léonie Island (67.5984°S, 68.3561°W) in the sub‐Antarctic, low maritime and high maritime Antarctic respectively. In order to achieve consistency between islands, soil was collected from under populations of co‐occurring *Deschampsia antarctica* Desv. and *Colobanthus quitensis* (Kunth) Bartl., the only two native vascular plant species that occur in Antarctica. On each island, 50 ml sterile centrifuge tubes (Corning Inc, Corning, NY, USA) were used to collect soil samples by hammering them directly into the vertical walls of three pits at three depths (2, 4 and 8 cm). Thus, a total of 27 soil samples were collected from across the three islands. The three pits were a maximum of 1 km apart on each island, with an average distance of 311 m separating them. The soil, kept on ice after collection and frozen at −80 **°**C within 5 h, was freeze dried to preserve fungal nucleotides.

### *Nucleic acid extraction and amplification*

Total DNA and RNA were extracted simultaneously from five individual 50 mg samples, taken from each of the tubes of homogenized soil (representing a total of 27 × 5 = 135 samples), using RNA PowerSoil Total RNA Isolation and DNA Elution Accessory kits (MoBio Laboratories, Carlsbad, CA, USA). Extracted DNA was amplified in triplicate PCR reactions using the primers ITS1F and ITS4 as described by Cox *et al*. (2016), with conditions matching those described below for cDNA. Extracted RNA was treated with a Turbo DNA‐free kit (Life technologies, Carlsbad, CA, USA), checked for the absence of DNA using PCR, and reverse transcribed using AccuScript High‐Fidelity Reverse Transcriptase (Agilent, Santa Clara, CA, USA) and random nonamers. The resulting cDNA was amplified in triplicate PCR reactions using ITS1F (Gardes and Bruns, 1993) and ITS4 (White *et al*., 1990) primers. The ITS4 primer was modified with the Roche 454 A adapter and a 10 bp barcode specific to each sample, allowing identification of different samples once pooled, and the ITS1F primer was modified with the 454 B adaptor.

The triplicate PCR products were pooled and subsequently purified using AMPure XP bead purification (Beckman Coulter, Inc, Brea, CA, USA) and quantified using a Qubit dsDNA HS Assay (Life Technologies, Carlsbad, CA, USA) before normalization to consistent concentration. The purified and normalized PCR products were run on a single plate, on the 454 Roche Titanium FLX platform at the Liverpool Centre for Genomic Research, at the same time and under identical conditions to the DNA library.

### *Sequence analyses*

The resulting RNA sequences were pooled with the DNA sequences (Cox *et al*., 2016) and processed together using the QIIME pipeline (Caporaso *et al*., 2010). Sequences were filtered to remove reads that were of low quality, less than 300 bp or greater than 1200 bp, and were split according to barcodes. The remaining sequences were denoised to reduce the influence of characteristic errors associated with 454 pyrosequencing, using the denoiser algorithm available in QIIME (Reeder and Knight, 2010), and checked for potential chimeras using UCHIME (Edgar *et al*., 2011). Abundant sequences flagged as potential chimeras through either denovo or reference based searches were manually checked, and confirmed chimeric sequences filtered from the dataset. As the ITS1 region was not always fully sequenced, the ITS2 regions of the remaining high quality non chimeric sequences were extracted using ITSx (Bengtsson‐Palme *et al*., 2013) to remove flanking conserved regions that can interfere with downstream sequence clustering. The ITS2 sequences were grouped into Operational Taxonomic Units (OTUs) at 97% sequence similarity using USEARCH 6.1 (Edgar, 2010), approximating to species‐level groupings. OTUs represented by a single sequence were subsequently removed from the analyses, since they may often represent erroneous sequences. Taxonomy was assigned to OTUs in QIIME, by running the BLAST algorithm against the UNITE fungal database, uploaded on 20 November 2016. RNA sequences were deposited in the NCBI Sequence Read Archive (accession PRJNA518063).

Sequences were BLAST‐searched (97%) against a previously described global database of 32 376 soil fungal ITS2 sequences (Cox *et al*., 2016). Briefly, this database was compiled from 14 studies focused on fungi occurring in soils at 394 sites around the world, with a minimum sequencing depth of 1000 sequences per site. The ITS2 region of sequences from all studies was extracted and clustered at 97% similarity using USEARCH 6.1 (Edgar, 2010), as implemented in QIIME (see Cox *et al*., 2016 for more details). These search results were used to assign each fungal OTU as either being endemic to Antarctica, or cosmopolitan if it was found at sites elsewhere. An OTU was classified as bipolar if it occurred north of the Arctic circle (66.56°N), but did not occur at latitudes in between. The taxonomic assignments of the endemic, cosmopolitan and bipolar fungi are shown in Supporting Information Table S1.

### *Statistical analyses*

The five replicates from each soil tube were pooled and measures of fungal richness, evenness, taxonomic diversity and taxonomic distinctness (Clarke and Warwick, 2001) were calculated for each DNA/RNA pair. Linear mixed effects models were used to compare these metrics between pairs of total and active samples, with island and sampling pit included as random effects. A linear mixed effects model was also used to test whether the magnitude of differences in fungal richness between active and total communities changed with soil depth.

The R function Adonis (permutational MANOVA) (Oksanen *et al*., 2017), was used to compare samples at the same depth within each pit, testing whether active community replicates were more similar to each other than they were to total community replicates, and *vice versa*. A generalized linear mixed effects model was used to test whether the proportion of fungi shared between paired samples of total and active communities changed with soil depth.

Permutational paired sign tests (using a custom VB script) were used to investigate whether endemic, cosmopolitan and bipolar fungi differed between total and active communities. This test was designed to identify differential abundances greater than the expected stochastic differences between sequencing runs. Based on BLAST searches against the global database of soil fungal ITS2 sequences (Cox *et al*., 2016) each Antarctic OTU was labelled as endemic, cosmopolitan or bipolar. The test statistic was calculated as the proportion of OTUs in each of these three collective groups with higher overall abundance in the relevant community (either total or active, since this was a two‐tailed test). This test statistic ensures that each OTU is weighted equally, and identifies consistent differences in abundance between communities. Alternative statistics based on differences in sequence reads or OTU richness of each community were considered to give undue weight to very abundant fungi with large numbers of reads, or rare fungi, which are disproportionately more likely to appear in one library but not the other respectively. Significance was tested by comparing observed test statistics to a distribution of 1000 null values, generated by randomizing labels (endemic/cosmopolitan/bipolar) across all OTUs. As we were assigning cosmopolitan status based on matches to a database of fungi detected using DNA‐based analyses, it is possible that sequences detected using only RNA were more likely to be assigned endemic status. Therefore, the results presented are for tests carried out on only fungi that were detected in both nucleic acid libraries; however, the results were qualitatively similar if all fungi were included in the test. The same tests were also used to investigate whether OTUs in different taxonomic classes differed in abundance between the two communities.

We used several approaches to ensure that differences in sequencing depth did not affect the above analyses. For comparisons of metrics based on species richness, communities were rarefied to the lowest common sequencing depth. The process of rarefaction has recently attracted criticism in the context of comparisons of community composition, due to the inherent loss of data involved (McMurdie and Holmes, 2014). In the context of this study, rarefying can be considered conservative, as the loss of data reduces the power to detect differences in abundance between DNA and RNA libraries (McMurdie and Holmes, 2014). All tests were carried out on 1000 iterations of the rarefied community matrix, and also repeated on the unrarefied dataset. For comparisons of community composition, Bray–Curtis distances were calculated from unrarefied, proportional transformed data (Legendre and Gallagher, 2001), and repeated on rarefied data. All conclusions were robust regardless of whether or not the data was rarefied – for comparisons of species richness, evenness and taxonomic distinctness that are dependent on equal sampling we present results from rarefied datasets; whilst for comparisons of community composition, we present results from unrarefied, normalized data.

## Supporting information


**Fig. S1.** Correlation between OTU richness of total (DNA) and active (RNA) samples. *R* (Pearson's) = 0.643, *n* = 27. 1:1 line is shown in red.
**Table S1.** The number of OTUs of each fungal genus, assigned as endemic, cosmopolitan or bipolar when compared to a global dataset of soil fungi. Taxonomies were assigned by running representative sequences through the BLAST algorithm against the UNITE fungal database. Bipolar fungi were defined as those occurring in Antarctica, and north of the Arctic circle (approximately 66.5° north), but not occurring at latitudes in between.
