# Supplementary material for: Endemic and cosmopolitan fungal taxa exhibit differential abundances in total and active communities of Antarctic soils
Source: Environ Microbiol. 2019 Feb 22;21(5):1586–96. doi: 10.1111/1462-2920.14533 (PMC6850668; doi:10.1111/1462-2920.14533)
Supplement: Supplementary file 1 — Fig. S1. Correlation between OTU richness of total (DNA) and active (RNA) samples. R (Pearson's) = 0.643, n = 27. 1:1 line is shown in red. Table S1. The number of OTUs of each fungal genus, assigned as endemic, cosmopolitan or bipolar when compared to a global dataset of soil fungi. Taxonomies were assigned by running representative sequences through the BLAST algorithm against the UNITE fungal database. Bipolar fungi were defined as those occurring in Antarctica, and north of the Arctic circle (approximately 66.5° north), but not occurring at latitudes in between. [file EMI-21-1586-s001.docx]

# Supplementary Information

**Figure S1** Correlation between OTU richness of total (DNA) and active (RNA) samples. R (Pearson’s)=0.643, n=27. 1:1 line is shown in red.

**Table S1** The number of OTUs of each fungal genus, assigned as endemic, cosmopolitan or bipolar when compared to a global dataset of soil fungi. Taxonomies were assigned by running representative sequences through the BLAST algorithm against the UNITE fungal database. Bipolar fungi were defined as those occurring in Antarctica, and north of the Arctic circle (approximately 66.5° north), but not occurring at latitudes in between.

|  |  |  |  |  | **Number of OTUs** | | |
| --- | --- | --- | --- | --- | --- | --- | --- |
| **Phylum** | **Class** | **Order** | **Family** | **Genus** | **Cosmopolitan** | **Endemic** | **Bipolar** |
| Ascomycota | Archaeorhizomycetes | Archaeorhizomycetales | Archaeorhizomycetaceae | *Archaeorhizomyces* | 0 | 1 | 0 |
| Ascomycota | Dothideomycetes | Botryosphaeriales | Unidentified Botryosphaeriales | Unidentified *Botryosphaeriales* | 0 | 1 | 0 |
| Ascomycota | Dothideomycetes | Capnodiales | Capnodiales *Incertae sedis* | *Capnobotryella* | 0 | 1 | 0 |
| Ascomycota | Dothideomycetes | Capnodiales | Capnodiales *Incertae sedis* | *Elasticomyces* | 0 | 1 | 0 |
| Ascomycota | Dothideomycetes | Capnodiales | Extremaceae | *Extremus* | 1 | 0 | 0 |
| Ascomycota | Dothideomycetes | Capnodiales | Mycosphaerellaceae | *Dissoconium* | 0 | 1 | 0 |
| Ascomycota | Dothideomycetes | Capnodiales | Mycosphaerellaceae | *Mycosphaerella* | 1 | 2 | 0 |
| Ascomycota | Dothideomycetes | Capnodiales | Mycosphaerellaceae | *Ramularia* | 0 | 1 | 0 |
| Ascomycota | Dothideomycetes | Capnodiales | Teratosphaeriaceae | *Neodevriesia* | 1 | 0 | 0 |
| Ascomycota | Dothideomycetes | Capnodiales | Teratosphaeriaceae | *Oleoguttula* | 0 | 1 | 0 |
| Ascomycota | Dothideomycetes | Capnodiales | Unidentified Capnodiales | Unidentified *Capnodiales* | 0 | 2 | 0 |
| Ascomycota | Dothideomycetes | Dothideales | Dothioraceae | *Aureobasidium* | 1 | 0 | 0 |
| Ascomycota | Dothideomycetes | Dothideales | Dothioraceae | *Kabatiella* | 0 | 1 | 0 |
| Ascomycota | Dothideomycetes | Dothideales | Unidentified Dothideales | Unidentified *Dothideales* | 0 | 1 | 0 |
| Ascomycota | Dothideomycetes | Dothideomycetes *Incertae sedis* | Dothideomycetes *Incertae sedis* | *Zymoseptoria* | 0 | 1 | 0 |
| Ascomycota | Dothideomycetes | Dothideomycetidae *Incertae sedis* | Parmulariaceae | *Parmularia* | 0 | 1 | 0 |
| Ascomycota | Dothideomycetes | Pleosporales | Didymellaceae | *Neoascochyta* | 2 | 2 | 0 |
| Ascomycota | Dothideomycetes | Pleosporales | Leptosphaeriaceae | *Coniothyrium* | 0 | 1 | 0 |
| Ascomycota | Dothideomycetes | Pleosporales | Leptosphaeriaceae | *Leptosphaeria* | 1 | 0 | 0 |
| Ascomycota | Dothideomycetes | Pleosporales | Massarinaceae | *Keissleriella* | 0 | 1 | 0 |
| Ascomycota | Dothideomycetes | Pleosporales | Phaeosphaeriaceae | *Ophiosphaerella* | 0 | 1 | 0 |
| Ascomycota | Dothideomycetes | Pleosporales | Phaeosphaeriaceae | *Phaeosphaeria* | 2 | 1 | 1 |
| Ascomycota | Dothideomycetes | Pleosporales | Phaeosphaeriaceae | *Sclerostagonospora* | 2 | 0 | 0 |
| Ascomycota | Dothideomycetes | Pleosporales | Phaeosphaeriaceae | Unidentified *Phaeosphaeriaceae* | 0 | 2 | 0 |
| Ascomycota | Dothideomycetes | Pleosporales | Pleosporaceae | *Alternaria* | 0 | 1 | 0 |
| Ascomycota | Dothideomycetes | Pleosporales | Pleosporaceae | Unidentified *Pleosporaceae* | 0 | 3 | 0 |
| Ascomycota | Dothideomycetes | Pleosporales | Pleosporales *Incertae sedis* | *Herpotrichia* | 1 | 0 | 0 |
| Ascomycota | Dothideomycetes | Pleosporales | Unidentified Pleosporales | Unidentified *Pleosporales* | 2 | 2 | 0 |
| Ascomycota | Dothideomycetes | Unidentified Dothideomycetes | Unidentified Dothideomycetes | Unidentified *Dothideomycetes* | 0 | 3 | 1 |
| Ascomycota | Dothideomycetes | Venturiales | Venturiaceae | *Venturia* | 1 | 0 | 0 |
| Ascomycota | Eurotiomycetes | Chaetothyriales | Chaetothyriaceae | *Cyphellophora* | 0 | 1 | 0 |
| Ascomycota | Eurotiomycetes | Chaetothyriales | Herpotrichiellaceae | *Capronia* | 0 | 1 | 0 |
| Ascomycota | Eurotiomycetes | Chaetothyriales | Herpotrichiellaceae | *Cladophialophora* | 1 | 7 | 1 |
| Ascomycota | Eurotiomycetes | Chaetothyriales | Herpotrichiellaceae | *Exophiala* | 2 | 0 | 0 |
| Ascomycota | Eurotiomycetes | Chaetothyriales | Herpotrichiellaceae | *Phaeococcomyces* | 0 | 1 | 0 |
| Ascomycota | Eurotiomycetes | Chaetothyriales | Herpotrichiellaceae | *Rhinocladiella* | 0 | 0 | 1 |
| Ascomycota | Eurotiomycetes | Chaetothyriales | Herpotrichiellaceae | Unidentified *Herpotrichiellaceae* | 2 | 11 | 1 |
| Ascomycota | Eurotiomycetes | Chaetothyriales | Unidentified Chaetothyriales | Unidentified *Chaetothyriales* | 1 | 5 | 0 |
| Ascomycota | Eurotiomycetes | Eurotiales | Trichocomaceae | *Aspergillus* | 4 | 0 | 0 |
| Ascomycota | Eurotiomycetes | Eurotiales | Trichocomaceae | *Paecilomyces* | 1 | 0 | 0 |
| Ascomycota | Eurotiomycetes | Eurotiales | Trichocomaceae | *Penicillium* | 3 | 0 | 0 |
| Ascomycota | Eurotiomycetes | Eurotiales | Unidentified Eurotiales | Unidentified *Eurotiales* | 0 | 2 | 0 |
| Ascomycota | Eurotiomycetes | Onygenales | Arthrodermataceae | *Ctenomyces* | 0 | 1 | 0 |
| Ascomycota | Eurotiomycetes | Onygenales | Onygenaceae | *Chrysosporium* | 0 | 1 | 0 |
| Ascomycota | Eurotiomycetes | Onygenales | Unidentified Onygenales | Unidentified *Onygenales* | 0 | 1 | 0 |
| Ascomycota | Eurotiomycetes | Verrucariales | Unidentified Verrucariales | Unidentified *Verrucariales* | 0 | 1 | 0 |
| Ascomycota | Eurotiomycetes | Verrucariales | Verrucariaceae | *Verrucaria* | 0 | 1 | 0 |
| Ascomycota | Lecanoromycetes | Acarosporales | Acarosporaceae | *Acarospora* | 0 | 1 | 0 |
| Ascomycota | Lecanoromycetes | Acarosporales | Acarosporaceae | Unidentified *Acarosporaceae* | 0 | 1 | 0 |
| Ascomycota | Lecanoromycetes | Candelariales | Candelariaceae | *Candelaria* | 0 | 1 | 0 |
| Ascomycota | Lecanoromycetes | Candelariales | Candelariaceae | *Candelariella* | 0 | 1 | 0 |
| Ascomycota | Lecanoromycetes | Lecanorales | Cladoniaceae | *Cladonia* | 0 | 2 | 0 |
| Ascomycota | Lecanoromycetes | Lecanorales | Haematommataceae | *Haematomma* | 0 | 1 | 0 |
| Ascomycota | Lecanoromycetes | Lecanorales | Lecanoraceae | *Lecidella* | 0 | 1 | 0 |
| Ascomycota | Lecanoromycetes | Lecanorales | Lecanoraceae | *Rhizoplaca* | 0 | 1 | 0 |
| Ascomycota | Lecanoromycetes | Lecanorales | Lecanoraceae | Unidentified *Lecanoraceae* | 0 | 1 | 0 |
| Ascomycota | Lecanoromycetes | Lecanorales | Lecanorales *Incertae sedis* | Unidentified *Lecanorales Incertae sedis* | 0 | 1 | 0 |
| Ascomycota | Lecanoromycetes | Lecanorales | Parmeliaceae | Unidentified *Parmeliaceae* | 0 | 1 | 0 |
| Ascomycota | Lecanoromycetes | Lecanorales | Parmeliaceae | *Usnea* | 0 | 1 | 0 |
| Ascomycota | Lecanoromycetes | Lecanorales | Ramalinaceae | *Bacidina* | 0 | 1 | 0 |
| Ascomycota | Lecanoromycetes | Lecanorales | Ramalinaceae | Unidentified *Ramalinaceae* | 0 | 1 | 0 |
| Ascomycota | Lecanoromycetes | Lecanorales | Ramalinaceae | *Waynea* | 0 | 1 | 0 |
| Ascomycota | Lecanoromycetes | Lecanorales | Stereocaulaceae | *Lepraria* | 0 | 2 | 1 |
| Ascomycota | Lecanoromycetes | Lecanorales | Unidentified Lecanorales | Unidentified *Lecanorales* | 0 | 1 | 0 |
| Ascomycota | Lecanoromycetes | Ostropales | Stictidaceae | *Absconditella* | 0 | 1 | 0 |
| Ascomycota | Lecanoromycetes | Peltigerales | Pannariaceae | Unidentified *Pannariaceae* | 0 | 1 | 0 |
| Ascomycota | Lecanoromycetes | Pertusariales | Megasporaceae | *Aspicilia* | 0 | 1 | 0 |
| Ascomycota | Lecanoromycetes | Pertusariales | Megasporaceae | Unidentified *Megasporaceae* | 0 | 2 | 0 |
| Ascomycota | Lecanoromycetes | Pertusariales | Pertusariaceae | *Loxosporopsis* | 0 | 1 | 0 |
| Ascomycota | Lecanoromycetes | Rhizocarpales | Rhizocarpaceae | *Rhizocarpon* | 0 | 1 | 0 |
| Ascomycota | Lecanoromycetes | Teloschistales | Physciaceae | *Buellia* | 0 | 3 | 0 |
| Ascomycota | Lecanoromycetes | Teloschistales | Physciaceae | *Phaeophyscia* | 0 | 1 | 0 |
| Ascomycota | Lecanoromycetes | Teloschistales | Physciaceae | *Physcia* | 0 | 1 | 0 |
| Ascomycota | Lecanoromycetes | Teloschistales | Teloschistaceae | *Caloplaca* | 0 | 1 | 0 |
| Ascomycota | Lecanoromycetes | Teloschistales | Teloschistaceae | Unidentified *Teloschistaceae* | 0 | 1 | 0 |
| Ascomycota | Lecanoromycetes | Teloschistales | Teloschistaceae | *Variospora* | 0 | 1 | 0 |
| Ascomycota | Lecanoromycetes | Teloschistales | Unidentified Teloschistales | Unidentified *Teloschistales* | 0 | 2 | 0 |
| Ascomycota | Lecanoromycetes | Umbilicariales | Umbilicariaceae | *Umbilicaria* | 0 | 2 | 0 |
| Ascomycota | Lecanoromycetes | Unidentified Lecanoromycetes | Unidentified Lecanoromycetes | Unidentified *Lecanoromycetes* | 0 | 3 | 0 |
| Ascomycota | Leotiomycetes | Helotiales | Dermateaceae | *Mollisia* | 1 | 0 | 0 |
| Ascomycota | Leotiomycetes | Helotiales | Helotiaceae | *Articulospora* | 1 | 0 | 0 |
| Ascomycota | Leotiomycetes | Helotiales | Helotiaceae | *Crocicreas* | 1 | 1 | 0 |
| Ascomycota | Leotiomycetes | Helotiales | Helotiaceae | *Hymenoscyphus* | 1 | 2 | 0 |
| Ascomycota | Leotiomycetes | Helotiales | Helotiaceae | *Neobulgaria* | 1 | 0 | 0 |
| Ascomycota | Leotiomycetes | Helotiales | Helotiaceae | *Rhizoscyphus* | 2 | 13 | 1 |
| Ascomycota | Leotiomycetes | Helotiales | Helotiales *Incertae sedis* | *Cadophora* | 2 | 0 | 0 |
| Ascomycota | Leotiomycetes | Helotiales | Helotiales *Incertae sedis* | *Chlorencoelia* | 0 | 2 | 0 |
| Ascomycota | Leotiomycetes | Helotiales | Helotiales *Incertae sedis* | *Cystodendron* | 1 | 0 | 0 |
| Ascomycota | Leotiomycetes | Helotiales | Helotiales *Incertae sedis* | *Glarea* | 0 | 1 | 0 |
| Ascomycota | Leotiomycetes | Helotiales | Helotiales *Incertae sedis* | *Leptodontidium* | 2 | 3 | 0 |
| Ascomycota | Leotiomycetes | Helotiales | Helotiales *Incertae sedis* | *Naevala* | 1 | 0 | 0 |
| Ascomycota | Leotiomycetes | Helotiales | Helotiales *Incertae sedis* | *Rhexocercosporidium* | 0 | 0 | 1 |
| Ascomycota | Leotiomycetes | Helotiales | Helotiales *Incertae sedis* | *Spirosphaera* | 0 | 2 | 0 |
| Ascomycota | Leotiomycetes | Helotiales | Helotiales *Incertae sedis* | *Tetracladium* | 0 | 1 | 0 |
| Ascomycota | Leotiomycetes | Helotiales | Hyaloscyphaceae | *Cistella* | 1 | 0 | 0 |
| Ascomycota | Leotiomycetes | Helotiales | Hyaloscyphaceae | *Hyaloscypha* | 1 | 0 | 0 |
| Ascomycota | Leotiomycetes | Helotiales | Hyaloscyphaceae | *Lachnum* | 0 | 1 | 0 |
| Ascomycota | Leotiomycetes | Helotiales | Hyaloscyphaceae | *Mycoarthris* | 0 | 1 | 0 |
| Ascomycota | Leotiomycetes | Helotiales | Hyaloscyphaceae | Unidentified *Hyaloscyphaceae* | 0 | 1 | 0 |
| Ascomycota | Leotiomycetes | Helotiales | Sclerotiniaceae | *Botrytis* | 1 | 0 | 0 |
| Ascomycota | Leotiomycetes | Helotiales | Unidentified Helotiales | Unidentified *Helotiales* | 15 | 20 | 1 |
| Ascomycota | Leotiomycetes | Helotiales | Vibrisseaceae | *Phialocephala* | 0 | 1 | 0 |
| Ascomycota | Leotiomycetes | Leotiales | Leotiaceae | *Alatospora* | 0 | 1 | 0 |
| Ascomycota | Leotiomycetes | Leotiales | Leotiaceae | *Pezoloma* | 3 | 3 | 0 |
| Ascomycota | Leotiomycetes | Leotiomycetes *Incertae sedis* | Leotiomycetes *Incertae sedis* | *Collophora* | 0 | 1 | 0 |
| Ascomycota | Leotiomycetes | Leotiomycetes *Incertae sedis* | Leotiomycetes *Incertae sedis* | *Geomyces* | 0 | 2 | 0 |
| Ascomycota | Leotiomycetes | Leotiomycetes *Incertae sedis* | Leotiomycetes *Incertae sedis* | *Meliniomyces* | 0 | 6 | 0 |
| Ascomycota | Leotiomycetes | Leotiomycetes *Incertae sedis* | Myxotrichaceae | *Oidiodendron* | 0 | 2 | 0 |
| Ascomycota | Leotiomycetes | Leotiomycetes *Incertae sedis* | Myxotrichaceae | *Pseudogymnoascus* | 3 | 1 | 0 |
| Ascomycota | Leotiomycetes | Leotiomycetes *Incertae sedis* | Myxotrichaceae | Unidentified *Myxotrichaceae* | 1 | 0 | 0 |
| Ascomycota | Leotiomycetes | Rhytismatales | Rhytismataceae | Unidentified *Rhytismataceae* | 0 | 1 | 0 |
| Ascomycota | Leotiomycetes | Thelebolales | Thelebolaceae | *Antarctomyces* | 0 | 1 | 0 |
| Ascomycota | Leotiomycetes | Thelebolales | Thelebolaceae | *Thelebolus* | 1 | 0 | 0 |
| Ascomycota | Leotiomycetes | Unidentified Leotiomycetes | Unidentified Leotiomycetes | Unidentified *Leotiomycetes* | 5 | 4 | 0 |
| Ascomycota | Lichinomycetes | Lichinales | Lichinaceae | *Phylliscum* | 0 | 1 | 0 |
| Ascomycota | Orbiliomycetes | Orbiliales | Orbiliaceae | *Arthrobotrys* | 0 | 1 | 0 |
| Ascomycota | Orbiliomycetes | Orbiliales | Orbiliaceae | *Brachyphoris* | 0 | 1 | 0 |
| Ascomycota | Orbiliomycetes | Orbiliales | Orbiliaceae | *Hyalorbilia* | 0 | 1 | 0 |
| Ascomycota | Orbiliomycetes | Orbiliales | Orbiliaceae | *Orbilia* | 0 | 1 | 0 |
| Ascomycota | Orbiliomycetes | Orbiliales | Orbiliaceae | Unidentified *Orbiliaceae* | 0 | 0 | 1 |
| Ascomycota | Orbiliomycetes | Orbiliales | Unidentified Orbiliales | Unidentified *Orbiliales* | 0 | 2 | 0 |
| Ascomycota | Orbiliomycetes | Unidentified Orbiliomycetes | Unidentified Orbiliomycetes | Unidentified *Orbiliomycetes* | 0 | 6 | 0 |
| Ascomycota | Pezizomycetes | Pezizales | Pyronemataceae | *Cheilymenia* | 1 | 0 | 0 |
| Ascomycota | Pezizomycotina *Incertae sedis* | Pezizomycotina *Incertae sedis* | Pezizomycotina *Incertae sedis* | *Calcarisporiella* | 0 | 1 | 0 |
| Ascomycota | Pezizomycotina *Incertae sedis* | Pezizomycotina *Incertae sedis* | Pezizomycotina *Incertae sedis* | *Chalara* | 1 | 0 | 0 |
| Ascomycota | Pezizomycotina *Incertae sedis* | Pezizomycotina *Incertae sedis* | Pezizomycotina *Incertae sedis* | *Cordana* | 0 | 1 | 0 |
| Ascomycota | Pezizomycotina *Incertae sedis* | Pezizomycotina *Incertae sedis* | Pezizomycotina *Incertae sedis* | *Hemibeltrania* | 0 | 1 | 0 |
| Ascomycota | Pezizomycotina *Incertae sedis* | Pezizomycotina *Incertae sedis* | Pezizomycotina *Incertae sedis* | *Infundibulomyces* | 0 | 1 | 0 |
| Ascomycota | Pezizomycotina *Incertae sedis* | Pezizomycotina *Incertae sedis* | Pezizomycotina *Incertae sedis* | *Knufia* | 1 | 0 | 0 |
| Ascomycota | Pezizomycotina *Incertae sedis* | Pezizomycotina *Incertae sedis* | Pezizomycotina *Incertae sedis* | *Periglandula* | 0 | 1 | 0 |
| Ascomycota | Pezizomycotina *Incertae sedis* | Pezizomycotina *Incertae sedis* | Pezizomycotina *Incertae sedis* | *Volucrispora* | 1 | 2 | 0 |
| Ascomycota | Pezizomycotina *Incertae sedis* | Pezizomycotina *Incertae sedis* | Pseudeurotiaceae | *Pseudeurotium* | 1 | 0 | 0 |
| Ascomycota | Pezizomycotina *Incertae sedis* | Pezizomycotina *Incertae sedis* | Pseudeurotiaceae | Unidentified *Pseudeurotiaceae* | 0 | 1 | 0 |
| Ascomycota | Saccharomycetes | Saccharomycetales | Pichiaceae | *Hyphopichia* | 0 | 1 | 0 |
| Ascomycota | Saccharomycetes | Saccharomycetales | Saccharomycetales *Incertae sedis* | *Botryozyma* | 0 | 1 | 0 |
| Ascomycota | Saccharomycetes | Saccharomycetales | Saccharomycetales *Incertae sedis* | *Candida* | 2 | 1 | 0 |
| Ascomycota | Saccharomycetes | Saccharomycetales | Saccharomycetales *Incertae sedis* | *Cyberlindnera* | 0 | 1 | 0 |
| Ascomycota | Saccharomycetes | Saccharomycetales | Saccharomycetales *Incertae sedis* | *Debaryomyces* | 1 | 0 | 0 |
| Ascomycota | Saccharomycetes | Saccharomycetales | Saccharomycetales *Incertae sedis* | *Kodamaea* | 0 | 1 | 0 |
| Ascomycota | Saccharomycetes | Saccharomycetales | Saccharomycetales *Incertae sedis* | *Peterozyma* | 0 | 1 | 0 |
| Ascomycota | Sordariomycetes | Chaetosphaeriales | Chaetosphaeriaceae | *Chaetosphaeria* | 1 | 0 | 0 |
| Ascomycota | Sordariomycetes | Chaetosphaeriales | Chaetosphaeriaceae | *Neopseudolachnella* | 0 | 1 | 0 |
| Ascomycota | Sordariomycetes | Chaetosphaeriales | Unidentified Chaetosphaeriales | Unidentified *Chaetosphaeriales* | 0 | 1 | 0 |
| Ascomycota | Sordariomycetes | Coniochaetales | Coniochaetaceae | Unidentified *Coniochaetaceae* | 0 | 1 | 0 |
| Ascomycota | Sordariomycetes | Coniochaetales | Unidentified Coniochaetales | Unidentified *Coniochaetales* | 1 | 0 | 0 |
| Ascomycota | Sordariomycetes | Hypocreales | Bionectriaceae | *Clonostachys* | 1 | 0 | 0 |
| Ascomycota | Sordariomycetes | Hypocreales | Clavicipitaceae | *Harposporium* | 0 | 1 | 0 |
| Ascomycota | Sordariomycetes | Hypocreales | Clavicipitaceae | *Pochonia* | 2 | 0 | 0 |
| Ascomycota | Sordariomycetes | Hypocreales | Clavicipitaceae | Unidentified *Clavicipitaceae* | 1 | 0 | 0 |
| Ascomycota | Sordariomycetes | Hypocreales | Cordycipitaceae | *Cordyceps* | 0 | 2 | 0 |
| Ascomycota | Sordariomycetes | Hypocreales | Cordycipitaceae | *Isaria* | 0 | 1 | 0 |
| Ascomycota | Sordariomycetes | Hypocreales | Cordycipitaceae | *Lecanicillium* | 1 | 0 | 0 |
| Ascomycota | Sordariomycetes | Hypocreales | Cordycipitaceae | *Rotiferophthora* | 0 | 1 | 0 |
| Ascomycota | Sordariomycetes | Hypocreales | Cordycipitaceae | Unidentified *Cordycipitaceae* | 0 | 2 | 0 |
| Ascomycota | Sordariomycetes | Hypocreales | Hypocreaceae | *Trichoderma* | 0 | 1 | 0 |
| Ascomycota | Sordariomycetes | Hypocreales | Hypocreales *Incertae sedis* | *Acremonium* | 2 | 2 | 0 |
| Ascomycota | Sordariomycetes | Hypocreales | Hypocreales *Incertae sedis* | *Myrothecium* | 0 | 1 | 0 |
| Ascomycota | Sordariomycetes | Hypocreales | Nectriaceae | *Gibberella* | 2 | 0 | 0 |
| Ascomycota | Sordariomycetes | Hypocreales | Nectriaceae | Unidentified *Nectriaceae* | 0 | 1 | 0 |
| Ascomycota | Sordariomycetes | Hypocreales | Ophiocordycipitaceae | *Haptocillium* | 0 | 5 | 0 |
| Ascomycota | Sordariomycetes | Hypocreales | Ophiocordycipitaceae | *Ophiocordyceps* | 0 | 1 | 0 |
| Ascomycota | Sordariomycetes | Hypocreales | Ophiocordycipitaceae | *Polycephalomyces* | 0 | 1 | 0 |
| Ascomycota | Sordariomycetes | Hypocreales | Ophiocordycipitaceae | *Tolypocladium* | 1 | 2 | 0 |
| Ascomycota | Sordariomycetes | Hypocreales | Unidentified Hypocreales | Unidentified *Hypocreales* | 1 | 9 | 0 |
| Ascomycota | Sordariomycetes | Microascales | Halosphaeriaceae | Unidentified *Halosphaeriaceae* | 1 | 0 | 0 |
| Ascomycota | Sordariomycetes | Microascales | Microascaceae | *Microascus* | 0 | 1 | 0 |
| Ascomycota | Sordariomycetes | Microascales | Microascaceae | Unidentified *Microascaceae* | 0 | 1 | 0 |
| Ascomycota | Sordariomycetes | Sordariales | Chaetomiaceae | *Chaetomium* | 0 | 1 | 0 |
| Ascomycota | Sordariomycetes | Sordariales | Chaetomiaceae | Unidentified *Chaetomiaceae* | 1 | 2 | 0 |
| Ascomycota | Sordariomycetes | Sordariales | Lasiosphaeriaceae | *Cercophora* | 1 | 2 | 0 |
| Ascomycota | Sordariomycetes | Sordariales | Lasiosphaeriaceae | *Podospora* | 0 | 1 | 0 |
| Ascomycota | Sordariomycetes | Sordariales | Lasiosphaeriaceae | Unidentified *Lasiosphaeriaceae* | 1 | 0 | 0 |
| Ascomycota | Sordariomycetes | Sordariales | Unidentified Sordariales | Unidentified *Sordariales* | 0 | 4 | 0 |
| Ascomycota | Sordariomycetes | Sordariomycetes *Incertae sedis* | Sordariomycetes *Incertae sedis* | *Myrmecridium* | 0 | 1 | 0 |
| Ascomycota | Sordariomycetes | Sordariomycetidae *Incertae sedis* | Glomerellaceae | *Colletotrichum* | 1 | 1 | 0 |
| Ascomycota | Sordariomycetes | Unidentified Sordariomycetes | Unidentified Sordariomycetes | Unidentified *Sordariomycetes* | 1 | 6 | 0 |
| Ascomycota | Sordariomycetes | Xylariales | Xylariales *Incertae sedis* | *Microdochium* | 1 | 0 | 0 |
| Ascomycota | Sordariomycetes | Xylariales | Xylariales *Incertae sedis* | *Monographella* | 2 | 0 | 0 |
| Ascomycota | Taphrinomycetes | Taphrinales | Protomycetaceae | *Protomyces* | 0 | 0 | 1 |
| Ascomycota | Taphrinomycetes | Taphrinales | Protomycetaceae | *Saitoella* | 0 | 3 | 0 |
| Ascomycota | Taphrinomycetes | Taphrinales | Taphrinaceae | *Taphrina* | 0 | 1 | 0 |
| Ascomycota | Unidentified Ascomycota | Unidentified Ascomycota | Unidentified Ascomycota | Unidentified *Ascomycota* | 7 | 33 | 2 |
| Basidiomycota | Agaricomycetes | Agaricales | Amanitaceae | *Amanita* | 0 | 1 | 0 |
| Basidiomycota | Agaricomycetes | Agaricales | Hygrophoraceae | Unidentified *Hygrophoraceae* | 0 | 1 | 0 |
| Basidiomycota | Agaricomycetes | Agaricales | Marasmiaceae | Unidentified *Marasmiaceae* | 0 | 1 | 0 |
| Basidiomycota | Agaricomycetes | Agaricales | Pleurotaceae | *Nematoctonus* | 1 | 0 | 0 |
| Basidiomycota | Agaricomycetes | Agaricales | Schizophyllaceae | *Schizophyllum* | 1 | 0 | 0 |
| Basidiomycota | Agaricomycetes | Agaricales | Strophariaceae | *Deconica* | 0 | 1 | 0 |
| Basidiomycota | Agaricomycetes | Agaricales | Strophariaceae | *Galerina* | 1 | 3 | 0 |
| Basidiomycota | Agaricomycetes | Agaricales | Strophariaceae | *Pholiota* | 0 | 1 | 0 |
| Basidiomycota | Agaricomycetes | Agaricales | Strophariaceae | *Psilocybe* | 0 | 1 | 0 |
| Basidiomycota | Agaricomycetes | Agaricales | Tricholomataceae | *Arrhenia* | 0 | 1 | 0 |
| Basidiomycota | Agaricomycetes | Agaricales | Tricholomataceae | Unidentified *Tricholomataceae* | 0 | 1 | 0 |
| Basidiomycota | Agaricomycetes | Agaricales | Unidentified Agaricales | Unidentified *Agaricales* | 1 | 0 | 1 |
| Basidiomycota | Agaricomycetes | Agaricomycetes *Incertae sedis* | Agaricomycetes *Incertae sedis* | *Peniophorella* | 0 | 1 | 0 |
| Basidiomycota | Agaricomycetes | Amylocorticiales | Amylocorticiaceae | *Amyloathelia* | 0 | 1 | 0 |
| Basidiomycota | Agaricomycetes | Atheliales | Atheliaceae | Unidentified *Atheliaceae* | 1 | 0 | 0 |
| Basidiomycota | Agaricomycetes | Auriculariales | Unidentified Auriculariales | Unidentified *Auriculariales* | 0 | 3 | 0 |
| Basidiomycota | Agaricomycetes | Cantharellales | Cantharellaceae | *Cantharellus* | 0 | 13 | 0 |
| Basidiomycota | Agaricomycetes | Cantharellales | Ceratobasidiaceae | Unidentified *Ceratobasidiaceae* | 0 | 2 | 0 |
| Basidiomycota | Agaricomycetes | Cantharellales | Clavulinaceae | Unidentified *Clavulinaceae* | 0 | 1 | 0 |
| Basidiomycota | Agaricomycetes | Cantharellales | Tulasnellaceae | *Tulasnella* | 0 | 1 | 0 |
| Basidiomycota | Agaricomycetes | Corticiales | Corticiaceae | *Corticium* | 0 | 1 | 0 |
| Basidiomycota | Agaricomycetes | Corticiales | Corticiaceae | *Laetisaria* | 0 | 1 | 0 |
| Basidiomycota | Agaricomycetes | Corticiales | Corticiaceae | *Marchandiobasidium* | 0 | 1 | 0 |
| Basidiomycota | Agaricomycetes | Hymenochaetales | Repetobasidiaceae | *Rickenella* | 0 | 1 | 0 |
| Basidiomycota | Agaricomycetes | Polyporales | Polyporaceae | *Trametes* | 0 | 1 | 0 |
| Basidiomycota | Agaricomycetes | Polyporales | Unidentified Polyporales | Unidentified *Polyporales* | 0 | 2 | 0 |
| Basidiomycota | Agaricomycetes | Russulales | Unidentified Russulales | Unidentified *Russulales* | 0 | 1 | 0 |
| Basidiomycota | Agaricomycetes | Sebacinales | Sebacinaceae | Unidentified *Sebacinaceae* | 0 | 1 | 0 |
| Basidiomycota | Agaricomycetes | Sebacinales | Sebacinales Group B | Unidentified *Sebacinales Group B* | 0 | 1 | 0 |
| Basidiomycota | Agaricomycetes | Sebacinales | Unidentified Sebacinales | Unidentified *Sebacinales* | 0 | 1 | 1 |
| Basidiomycota | Agaricomycetes | Trechisporales | Hydnodontaceae | *Trechispora* | 0 | 1 | 0 |
| Basidiomycota | Agaricomycetes | Unidentified Agaricomycetes | Unidentified Agaricomycetes | Unidentified *Agaricomycetes* | 0 | 3 | 0 |
| Basidiomycota | Agaricostilbomycetes | Agaricostilbales | Agaricostilbaceae | *Bensingtonia* | 0 | 1 | 0 |
| Basidiomycota | Agaricostilbomycetes | Agaricostilbales | Chionosphaeraceae | *Kurtzmanomyces* | 0 | 1 | 0 |
| Basidiomycota | Agaricostilbomycetes | Agaricostilbales | Kondoaceae | *Kondoa* | 0 | 2 | 0 |
| Basidiomycota | Agaricostilbomycetes | Agaricostilbales | Unidentified Agaricostilbales | Unidentified *Agaricostilbales* | 0 | 1 | 0 |
| Basidiomycota | Cystobasidiomycetes | Cystobasidiales | Cystobasidiaceae | *Cystobasidium* | 0 | 2 | 0 |
| Basidiomycota | Entorrhizomycetes | Entorrhizales | Entorrhizaceae | *Entorrhiza* | 0 | 2 | 0 |
| Basidiomycota | Exobasidiomycetes | Entylomatales | Unidentified Entylomatales | Unidentified *Entylomatales* | 0 | 1 | 0 |
| Basidiomycota | Exobasidiomycetes | Exobasidiomycetidae *Incertae sedis* | Exobasidiomycetidae *Incertae sedis* | *Meira* | 0 | 1 | 0 |
| Basidiomycota | Exobasidiomycetes | Exobasidiomycetidae *Incertae sedis* | Exobasidiomycetidae *Incertae sedis* | *Tilletiopsis* | 0 | 1 | 0 |
| Basidiomycota | Exobasidiomycetes | Unidentified Exobasidiomycetes | Unidentified Exobasidiomycetes | Unidentified *Exobasidiomycetes* | 0 | 1 | 0 |
| Basidiomycota | Microbotryomycetes | Leucosporidiales | Leucosporidiaceae | *Leucosporidium* | 1 | 1 | 0 |
| Basidiomycota | Microbotryomycetes | Leucosporidiales | Leucosporidiaceae | *Mastigobasidium* | 0 | 1 | 1 |
| Basidiomycota | Microbotryomycetes | Leucosporidiales | Unidentified Leucosporidiales | Unidentified *Leucosporidiales* | 3 | 6 | 0 |
| Basidiomycota | Microbotryomycetes | Microbotryales | Unidentified Microbotryales | Unidentified *Microbotryales* | 0 | 2 | 0 |
| Basidiomycota | Microbotryomycetes | Microbotryomycetes *Incertae sedis* | Chrysozymaceae | *Chrysozyma* | 0 | 5 | 0 |
| Basidiomycota | Microbotryomycetes | Microbotryomycetes *Incertae sedis* | Microbotryomycetes *Incertae sedis* | *Glaciozyma* | 0 | 1 | 0 |
| Basidiomycota | Microbotryomycetes | Sporidiobolales | Sporidiobolales *Incertae sedis* | *Rhodotorula* | 4 | 5 | 1 |
| Basidiomycota | Microbotryomycetes | Sporidiobolales | Sporidiobolales *Incertae sedis* | *Sporobolomyces* | 0 | 2 | 0 |
| Basidiomycota | Microbotryomycetes | Sporidiobolales | Unidentified Sporidiobolales | Unidentified *Sporidiobolales* | 0 | 2 | 0 |
| Basidiomycota | Microbotryomycetes | Unidentified Microbotryomycetes | Unidentified Microbotryomycetes | Unidentified *Microbotryomycetes* | 0 | 3 | 0 |
| Basidiomycota | Pucciniomycetes | Platygloeales | Eocronartiaceae | *Eocronartium* | 0 | 1 | 0 |
| Basidiomycota | Pucciniomycetes | Unidentified Pucciniomycetes | Unidentified Pucciniomycetes | Unidentified *Pucciniomycetes* | 0 | 1 | 0 |
| Basidiomycota | Tremellomycetes | Cystofilobasidiales | Cystofilobasidiaceae | *Mrakia* | 2 | 3 | 0 |
| Basidiomycota | Tremellomycetes | Cystofilobasidiales | Cystofilobasidiales *Incertae sedis* | *Mrakiella* | 1 | 1 | 0 |
| Basidiomycota | Tremellomycetes | Filobasidiales | Filobasidiaceae | *Filobasidium* | 2 | 0 | 0 |
| Basidiomycota | Tremellomycetes | Filobasidiales | Filobasidiaceae | *Goffeauzyma* | 1 | 0 | 0 |
| Basidiomycota | Tremellomycetes | Filobasidiales | Piskurozymaceae | *Solicoccozyma* | 2 | 0 | 0 |
| Basidiomycota | Tremellomycetes | Holtermanniales | Holtermanniales *Incertae sedis* | *Holtermanniella* | 1 | 1 | 0 |
| Basidiomycota | Tremellomycetes | Tremellales | Tremellales *Incertae sedis* | *Bullera* | 2 | 0 | 0 |
| Basidiomycota | Tremellomycetes | Tremellales | Tremellales *Incertae sedis* | *Cryptococcus* | 5 | 5 | 0 |
| Basidiomycota | Tremellomycetes | Tremellales | Tremellales *Incertae sedis* | *Dioszegia* | 0 | 4 | 2 |
| Basidiomycota | Tremellomycetes | Tremellales | Tremellales *Incertae sedis* | *Tremella* | 0 | 1 | 0 |
| Basidiomycota | Tremellomycetes | Tremellales | Tremellales *Incertae sedis* | Unidentified *Tremellales Incertae sedis* | 1 | 0 | 0 |
| Basidiomycota | Tremellomycetes | Tremellales | Unidentified Tremellales | Unidentified *Tremellales* | 0 | 4 | 0 |
| Basidiomycota | Tremellomycetes | Trichosporonales | Trichosporonaceae | *Apiotrichum* | 0 | 1 | 0 |
| Basidiomycota | Tremellomycetes | Trichosporonales | Trichosporonaceae | *Trichosporon* | 0 | 1 | 0 |
| Basidiomycota | Tremellomycetes | Unidentified Tremellomycetes | Unidentified Tremellomycetes | Unidentified *Tremellomycetes* | 0 | 7 | 0 |
| Basidiomycota | Unidentified Basidiomycota | Unidentified Basidiomycota | Unidentified Basidiomycota | Unidentified *Basidiomycota* | 3 | 18 | 0 |
| Basidiomycota | Ustilaginomycotina *Incertae sedis* | Malasseziales | Malasseziaceae | *Malassezia* | 1 | 1 | 0 |
| Basidiomycota | Ustilaginomycotina *Incertae sedis* | Malasseziales | Unidentified Malasseziales | Unidentified *Malasseziales* | 1 | 0 | 0 |
| Basidiomycota | Wallemiomycetes | Wallemiales | Wallemiaceae | *Wallemia* | 2 | 0 | 0 |
| Chytridiomycota | Chytridiomycetes | Chytridiales | Chytridiaceae | Unidentified *Chytridiaceae* | 0 | 3 | 0 |
| Chytridiomycota | Chytridiomycetes | Chytridiales | Endochytriaceae | *Entophlyctis* | 0 | 2 | 0 |
| Chytridiomycota | Chytridiomycetes | Chytridiomycetidae *Incertae sedis* | Chytridiomycetidae *Incertae sedis* | *Hyaloraphidium* | 0 | 2 | 0 |
| Chytridiomycota | Chytridiomycetes | Lobulomycetales | Lobulomycetaceae | *Clydaea* | 0 | 1 | 0 |
| Chytridiomycota | Chytridiomycetes | Lobulomycetales | Lobulomycetaceae | *Lobulomyces* | 0 | 1 | 0 |
| Chytridiomycota | Chytridiomycetes | Olpidiales | Unidentified Olpidiales | Unidentified *Olpidiales* | 0 | 1 | 0 |
| Chytridiomycota | Chytridiomycetes | Rhizophlyctidales | Rhizophlyctidaceae | *Rhizophlyctis* | 0 | 1 | 0 |
| Chytridiomycota | Chytridiomycetes | Rhizophlyctidales | Sonoraphlyctidaceae | *Sonoraphlyctis* | 0 | 1 | 0 |
| Chytridiomycota | Chytridiomycetes | Rhizophydiales | Alphamycetaceae | *Betamyces* | 0 | 2 | 0 |
| Chytridiomycota | Chytridiomycetes | Rhizophydiales | Protrudomycetaceae | *Protrudomyces* | 0 | 1 | 0 |
| Chytridiomycota | Chytridiomycetes | Rhizophydiales | Rhizophydiales *Incertae sedis* | *Halomyces* | 0 | 1 | 0 |
| Chytridiomycota | Chytridiomycetes | Rhizophydiales | Rhizophydiales *Incertae sedis* | *Operculomyces* | 0 | 1 | 0 |
| Chytridiomycota | Chytridiomycetes | Rhizophydiales | Unidentified Rhizophydiales | Unidentified *Rhizophydiales* | 0 | 1 | 0 |
| Chytridiomycota | Chytridiomycetes | Spizellomycetales | Spizellomycetaceae | *Spizellomyces* | 1 | 0 | 0 |
| Chytridiomycota | Chytridiomycetes | Spizellomycetales | Spizellomycetaceae | Unidentified *Spizellomycetaceae* | 0 | 1 | 0 |
| Chytridiomycota | Chytridiomycetes | Spizellomycetales | Unidentified Spizellomycetales | Unidentified *Spizellomycetales* | 0 | 2 | 0 |
| Chytridiomycota | Chytridiomycetes | Unidentified Chytridiomycetes | Unidentified Chytridiomycetes | Unidentified *Chytridiomycetes* | 0 | 5 | 0 |
| Chytridiomycota | Monoblepharidomycetes | Monoblepharidales | Unidentified Monoblepharidales | Unidentified *Monoblepharidales* | 0 | 2 | 0 |
| Chytridiomycota | Unidentified Chytridiomycota | Unidentified Chytridiomycota | Unidentified Chytridiomycota | Unidentified *Chytridiomycota* | 2 | 55 | 0 |
| Glomeromycota | Glomeromycetes | Archaeosporales | Archaeosporaceae | Unidentified *Archaeosporaceae* | 0 | 1 | 0 |
| Glomeromycota | Glomeromycetes | Glomerales | Glomeraceae | Unidentified *Glomeraceae* | 0 | 2 | 0 |
| Glomeromycota | Glomeromycetes | Unidentified Glomeromycetes | Unidentified Glomeromycetes | Unidentified *Glomeromycetes* | 0 | 3 | 0 |
| Rozellomycota | Unidentified Rozellomycota | Unidentified Rozellomycota | Unidentified Rozellomycota | Unidentified *Rozellomycota* | 6 | 27 | 1 |
| Unidentified fungi | Unidentified fungi | Unidentified fungi | Unidentified fungi | Unidentified *fungi* | 17 | 414 | 8 |
| Zygomycota | Mortierellomycotina *Incertae sedis* | Mortierellales | Mortierellaceae | *Dissophora* | 1 | 0 | 0 |
| Zygomycota | Mortierellomycotina *Incertae sedis* | Mortierellales | Mortierellaceae | *Mortierella* | 13 | 10 | 0 |
| Zygomycota | Mortierellomycotina *Incertae sedis* | Mortierellales | Unidentified Mortierellales | Unidentified *Mortierellales* | 0 | 2 | 0 |
| Zygomycota | Unidentified Zygomycota | Unidentified Zygomycota | Unidentified Zygomycota | Unidentified *Zygomycota* | 0 | 1 | 0 |
| Zygomycota | Zoopagomycotina *Incertae sedis* | Zoopagales | Piptocephalidaceae | *Kuzuhaea* | 0 | 1 | 0 |
| Zygomycota | Zoopagomycotina *Incertae sedis* | Zoopagales | Piptocephalidaceae | *Piptocephalis* | 0 | 1 | 0 |
| Zygomycota | Zygomycota *Incertae sedis* | Basidiobolales | Basidiobolaceae | *Basidiobolus* | 1 | 1 | 0 |
| Zygomycota | Zygomycota *Incertae sedis* | Basidiobolales | Basidiobolaceae | *Schizangiella* | 0 | 1 | 0 |
